# Study of the N-Terminal Domain Homodimerization in Human Proteins with Zinc Finger Clusters

**DOI:** 10.1134/S1607672921040050

**Published:** 2021-08-23

**Authors:** D. V. Fursenko, P. G. Georgiev, A. N. Bonchuk

**Affiliations:** Institute of Gene Biology, Russian Academy of Sciences, Moscow, Russia

**Keywords:** C2H2 proteins, dimerization, DUF3669, ZFP, transcription factor

## Abstract

CTCF belongs to a large family of transcription factors with clusters of C2H2-type zinc finger domains (C2H2 proteins) and is a main architectural protein in mammals. Human CTCF has a homodimerizing unstructured domain at the N-terminus which is involved in long-distance interactions. To test the presence of similar N-terminal domains in other human C2H2 proteins, a yeast two-hybrid system was used. In total, the ability of unstructured N-terminal domains to homodimerize was investigated for six human C2H2 proteins with an expression profile similar to CTCF. The data indicate the lack of the homodimerization ability of these domains. On the other hand, three C2H2 proteins containing the structured domain DUF3669 at the N-terminus demonstrated homo- and heterodimerization activity.

CTCF, the most studied mammalian architectural protein, consists of unstructured terminal regions and a cluster of 11 C2H2-type zinc fingers (C2H2 domains) located in the central part [1]. In human CTCF, C2H2 domains 3–7 are responsible for specific binding to the 15-bp consensus sequence [2]. The cluster of C2H2 domains is the only conserved part of the CTCF protein, which has a high level of homology in most vertebrates, insects, and some nematodes [3]. Proteins with clusters of five or more C2H2 domains (C2H2 proteins) are capable of specific recognition of extended DNA sequences and, in different taxa, constitute a significant part of DNA-binding transcription factors, the functions of which is still poorly understood [1, 4].

Among approximately 170 C2H2 proteins of *Drosophila*, many perform an architectural function, supporting long- distance interactions. The majority of them, in addition to the cluster of C2H2 domains, have structured domains at the N-terminus, which are capable of specific homodimerization [5]. It was shown that homodimerization of N-domains in such proteins is necessary to maintain long-distance interactions [4, 6]. We assume that the proportion of mammalian C2H2 proteins that perform an architectural function is much larger than it is currently believed and that the mechanism for maintaining long-distance interactions does not fundamentally differ between mammals and insects. However, only a relatively small part of mammalian C2H2 proteins contain structured N-terminal SCAN or BTB domains, which are capable of homodimerization [4]. BTB domains form predominantly stable homodimers [7], whereas SCAN domains are capable of homodimerization and selective heterodimerization [8]. Another poorly studied domain, DUF3669, was selectively shown to be capable of homo- and heterodimerization [9].

The  absence  of  the  characterized  dimerizing N-terminal domains in mammalian C2H2 proteins can be explained by the ability of unstructured domains to oligomerize, as we showed for the main human architectural protein CTCF and its orthologues [10]. To test this hypothesis, six C2H2 proteins whose expression pattern is similar to that of the CTCF protein were selected (Fig. 1). The analysis also included three C2H2 proteins with an N-terminal DUF3669 domain, for a more complete characterization of the dimerizing ability of this domain (Fig. 2a).

**Fig. 1.**
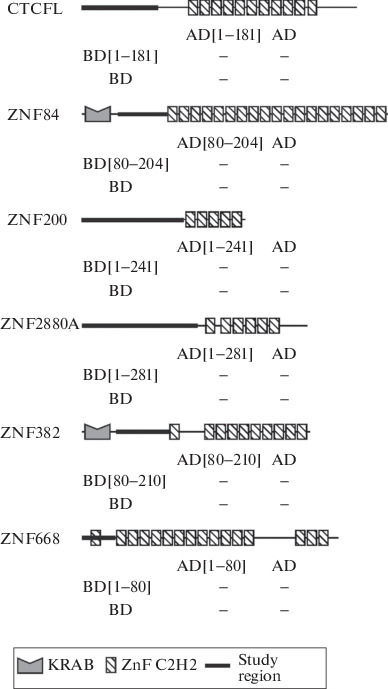
Investigation of the ability for homodimerization of the N-terminal regions of C2H2-containing human proteins in a yeast two-hybrid system. To test the ability for homodimerization, the ability of yeast co-expressing an N-terminal region linked to the GAL4 DNA-binding domain (BD) or the GAL4 activation domain (AD) to grow on a selective medium was observed. On the scheme, the domain structure of the proteins, the investigated region is indicated with a thick line, and the corresponding numbers of amino acid residues are indicated underneath. Below are the results of experiments with the yeast two-hybrid system, where signs + and – denote the presence or absence of interaction between the corresponding constructs. The ability for homodimerization of the N-terminus of the human CTCF protein (1–264 a.a.) was used as a positive control, and testing for the presence of interaction only with the activatory (AD) or DNA-binding (BD) domain of the GAL4 protein served as a negative control. Designations: KRAB—Krüppel-associated box domain, ZnF C2H2—C2H2-type zinc finger domain.

**Fig. 2.**
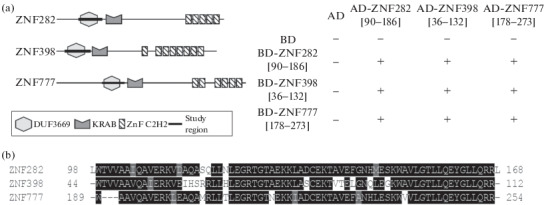
(a) Domain structures of DUF3669-containing proteins (left) and the results obtained in the yeast two-hybrid system (right). The results are presented similarly to Fig. 1. Designations: AD—activatory domain, BD—DNA-binding domain. (b) Alignment of amino acid sequences of the DUF3669 domains of human proteins ZNF282, ZNF398, and ZNF777. DUF3669—domain with unknown function 3669, KRAB—Krüppel-associated box domain, ZnF C2H2—C2H2-type zinc finger domain.

The ability of the N-terminal domains to undergo homodimerization was studied in a yeast two-hybrid system. Earlier, this method made it possible to identify the ability for homodimerization of the unstructured N-terminal region of the CTCF protein in different animal species [10]. In contrast to the unstructured N-terminal domain of human CTCF, the dimerization of which served as a positive control in this experiment, interactions between the N-terminal regions of proteins ZNF84, ZNF200, ZNF280A, ZNF382, ZNF668, as well as CTCFL, a paralog of CTCF, were found (Fig. 1). The last result is of particular interest, because CTCFL has significant similarity to CTCF in the structure of zinc fingers and can bind to its sites; however, it is expressed predominantly in mammalian testes [11]. It is assumed that CTCFL is involved in the regulation of the architectural functions of CTCF, replacing it in certain cases at sites and thereby disrupting the formation of chromatin loops.

In humans, seven proteins with the DUF3669 domain were predicted; however, only three of them (ZNF282, ZNF398, and ZNF777) have a cluster of five or more C2H2-type zinc fingers at the C terminus (Fig. 2a, left). These proteins are located in the same cluster in the genome of humans and many animal species, and the amino acid sequence of the DUF3669 domain of these proteins in humans has a high degree of homology (Fig. 2b). In addition to the high degree of similarity, in the yeast two-hybrid system, the DUF3669 domain of all C2H2 proteins studied in this work is capable of not only homo- but also heterodimerization (Fig. 2a, right).

The ability to specifically bind to long DNA motifs and to undergo homodimerization are the distinctive properties of architectural proteins of *Drosophila*. In mammals, out of ~800 C2H2 proteins, only 84 have dimerizing SCAN or BTB domains [1]. In our study, we confirmed and showed that the poorly studied N-terminal domain DUF3669 is also capable of forming homo- and heterodimers in three human C2H2 proteins. According to the model developed on *Drosophila*, it is assumed that the majority of C2H2 proteins are involved in the organization of chromosome architecture. The predominant homodimerization of the N-terminal domains of C2H2 proteins in *Droso-phila* is key in organizing specific long-distance interactions [12]. However, among the human C2H2 proteins, dimerization of N-terminal domains is spread insignificantly as compared to the analogous proteins of *Drosophila* [4]. In addition, the results of our work do not confirm the assumption that homodimerization due to the unstructured N-terminal region is widespread among the human C2H2 proteins. It is worth noting that the most studied architectural/insulator protein of *Drosophila* Su(Hw) at the N terminus also did not have a domain capable of homodimerization [13]. A possible, though poorly studied, mechanism of homodimerization for such proteins may be the involvement of C2H2 domains themselves in this process. For example, it was shown that the human YY1 protein is homodimerized with the involvement of the C2H2 domains [14]. Thus, further study of the mechanisms of homodimer formation in human C2H2 proteins is required.
